# Probiotics decrease C-reactive protein level in depression depending on basal chronic low-grade inflammation status or antidepressant use – secondary results of the Pro-demet randomized clinical trail

**DOI:** 10.1192/j.eurpsy.2025.2028

**Published:** 2025-08-26

**Authors:** O. Gawlik-Kotelnicka, A. Wysokiński, A. Gajewska, K. Czarnecka-Chrebelska, K. Kopacz, A. Skowrońska, E. Pikus, E. Brzeziańska-Lasota, D. Strzelecki

**Affiliations:** 1 Department of Affective and Psychotic Disorders; 2 Department of Old Age Psychiatry and Psychotic Disorders; 3 Faculty of Medicine; 4 Department of Biomedicine and Genetics; 5 ”Dynamo Lab” Academic Laboratory of Movement and Human Physical Performance, Medical University of Lodz, Lodz, Poland

## Abstract

**Introduction:**

There is a need to search for new treatment options not only for depression but also its concomitant diseases. Particularly, depression and metabolic-health abnormalities often coexist, while inflammation and microbiota imbalance, may play a part in their pathophysiological overlap. Thus, the trials of interventions on the microbiota may result in establishing a safe adjunctive treatment option.

**Objectives:**

The primary aim of this seconadry analysis was to assess the effect of probiotic formulation on inflammatory parameters in adult patients with depressive disorders. The secondary aim was to assess some possible pretreatment determinants of probiotics action towards inflammation, e.g., dietary habits, inflammatory or metabolic status, severity and dimensions of psychiatric symptoms, medications used.

**Methods:**

The parent trial was a two-arm, 60-day, prospective, randomized, double-blind, controlled design. The probiotic formulation contained Lactobacillus helveticus Rosell®-52 and Bifidobacterium longum Rosell®-175. The change in inflammatory parameters (e.g., C-reactive protein, complete blood count-derived markers, tumor necrosis factor-alpha) after intervention alone and in the context of basal lifestyle, psychometric, metabolic, and inflammatory parameters was assessed.

**Results:**

Probiotics significantly decreased CRP levels compared with placebo by 21.3 % (p = .047) with nearly moderate effect size as measured with Cliff’s delta (∆ = .249). Rates of CRP-responders (a minimum 50% decrease in CRP level) were non-significantly higher in the probiotic than placebo group (18.0 % vs. 5.26 %, respectively; χ2(1) = 3.20, p = .074); but the effect size was shown to be clinically meaningful (OR = 3.95; NNT = 7.85). In two-way ANOVA with interaction analysis, probiotics anti-inflammatory action was shown to be favoured by antidepressant use, and higher basal alanine aminotransferase. But, pretreatment chronic low-grade inflammation status counteracted probiotics anti-inflammatory properties.

There were no significant differences in complete blood count-derived parameters, nor in TNF-α levels.

**Conclusions:**

We have found an anti-inflammatory action of probiotics in patients with depression, as shown in the assessment of CRP levels. Additionally, probiotics were revealed to be more effective for inflammation measured by CRP levels when used by subjects with certain pretreatment features. Further studies should be performed to replicate these results.

ClinicalTrials.gov identifier: NCT04756544.

**Disclosure of Interest:**

None Declared

